# Enhancing radioactive iodine (RAI) incorporation in RAI-refractory differentiated thyroid cancer: current insights

**DOI:** 10.1530/ETJ-24-0319

**Published:** 2025-03-24

**Authors:** Tomo Hiromasa, Hiroshi Wakabayashi, Satoru Watanabe, Takafumi Yamase, Seigo Kinuya

**Affiliations:** Department of Nuclear Medicine, Kanazawa University Hospital, Kanazawa, Ishikawa, Japan

**Keywords:** enhancing RAI incorporation, RAI therapy, RAIR-DTC, TKI

## Abstract

Metastatic differentiated thyroid cancer (DTC) is responsible for most thyroid cancer-related deaths, with an even worse prognosis for patients with radioactive iodine (RAI)-refractory DTC (RAIR-DTC). While multikinase inhibitors (MKIs) and tyrosine kinase inhibitors (TKIs) offer effective treatments for RAIR-DTC, most patients remain noncurative and eventually experience disease progression. In addition, long-term use of these medications is hindered by adverse events, drug resistance and high cost. Recently, the use of MKIs and TKIs has reignited interest in enhancing RAI incorporation. This approach aims to restore the effectiveness of RAI therapy in patients with RAIR-DTC by using agents that increase RAI uptake, potentially overcoming current treatment challenges. This review covers the molecular mechanisms behind RAI resistance, the definition of RAIR-DTC and the efforts to enhance RAI incorporation through various agents, including those currently undergoing clinical trials.

## Introduction

Thyroid cancer is the most common endocrine malignancy, with a global incidence of 6.7 per 100,000 people (1). Differentiated thyroid cancer (DTC) accounts for approximately 90% of all thyroid cancers, including papillary thyroid carcinoma (PTC), follicular thyroid carcinoma (FTC), oncocytic carcinoma of the thyroid (OCA), invasive encapsulated follicular variant papillary thyroid carcinoma (IEFV-PTC) and poorly differentiated thyroid carcinoma (PDTC) (2). Depending on disease progression, the primary treatment strategy involves partial or total thyroidectomy, followed by RAI therapy. Risk stratification guides the indication for RAI therapy and the subsequent treatment approach (3). RAI therapy consists of three categories: ablation for low-risk groups, adjuvant therapy for moderate- to high-risk groups and RAI treatment for patients with residual disease or distant metastases. Approximately 10% of patients with DTC present with distant metastases, and 6–20% experience relapse at distant sites (4, 5). Overall, patients with DTC have an excellent 10-year disease-specific survival rate of about 95% (6), although distant metastatic DTC accounts for most thyroid cancer-related deaths. Therefore, RAI therapy for metastatic DTC aims to improve progression-free survival (PFS), disease-specific survival and overall survival (OS).

While RAI therapy has long been the standard treatment for patients with DTC and distant metastases, not all patients achieve satisfactory outcomes. The ^131^I used in RAI therapy emits both beta and gamma rays. The gamma rays help confirm RAI accumulation in tumor sites. However, around 40% of patients with distant metastases show RAI nonavidity during initial treatment (7, 8). Decreased RAI uptake can be either congenital or acquired during treatment (9), and eventually, more than half of patients develop resistance to RAI. Durante *et al.* (10) reported a 10-year survival rate of 92% for patients whose metastases were completely cured by RAI, compared to just 29% for those with residual disease. Whether RAI is avid or nonavid, the prognosis for patients with RAI resistance remains poor (11). Early development of RAI resistance is also a significant negative prognostic factor (12). Durante *et al.* (10) further noted that the 10-year OS after the diagnosis of metastatic disease was 56% in patients with RAI uptake, compared to just 10% in those without it. A recent systematic review confirmed that RAI nonavidity is an independent poor prognostic factor for both OS and PFS (13). Thus, RAI uptake status plays a critical prognostic role in patients with distant metastases. Given these challenges, aggressive therapeutic intervention for RAI-refractory DTC (RAIR-DTC) has become an urgent priority. In the past decade, multikinase inhibitors (MKIs) and tyrosine kinase inhibitors (TKIs) have emerged as post-RAI therapy options for RAIR-DTC.

Several unresolved issues persist with the use of MKIs and TKIs, despite their demonstrated efficacy in RAIR-DTC. MKIs such as lenvatinib and sorafenib, along with TKIs such as MEK and BRAF inhibitors, have shown favorable therapeutic outcomes in RAIR-DTC (14, 15, 16). However, even with these effective agents, most patients remain noncurative and eventually experience disease progression. The characteristic adverse events (AEs) of MKIs, including hypertension, diarrhea, fatigue, palmar-plantar erythrodysesthesia syndrome and rash, often necessitate dose reductions or discontinuation. A systematic review highlighted the therapeutic efficacy of lenvatinib but also noted the high incidence of grade 3 or higher AEs compared to other MKIs (17). The review reported a 78.6% incidence of grade 3 or higher AEs with lenvatinib, while serious AEs occurred in 37.2% of cases with sorafenib. Similarly, TKIs such as BRAF/MEK inhibitors commonly cause skin and subcutaneous tissue disorders, fever, hyperglycemia and fatigue, requiring careful management of side effects (16). A review of gliomas with *BRAF^V600^* mutations also concluded that although the treatment was effective, its toxicity severely limited tolerability (18). Moreover, these MKIs and TKIs are expensive, and financial constraints often hinder their long-term use. For instance, lenvatinib and sorafenib cost £52,307 and £38,746 per year, respectively. In the base-case economic analysis, the cost-effectiveness comparison of lenvatinib versus best supportive care results in an incremental cost-effectiveness ratio (ICER) of £65,872 per quality-adjusted life-year (QALY) gained, whereas that of sorafenib versus best supportive care results in an ICER of £85,644 per QALY gained (19). Furthermore, prolonged use of BRAF inhibitors can lead to drug resistance (20). Therefore, therapy response, AEs, cost and drug resistance are substantial barriers to the sustained use of MKIs and TKIs. Despite advances in new therapies over the past decade, the key challenge remains selecting the appropriate treatment at the right time and developing a sustainable treatment strategy for each individual.

The advent of MKIs and TKIs has reignited interest in enhancing RAI incorporation, also known as redifferentiation therapy. This approach aims to restore the effectiveness of RAI therapy in patients with RAIR-DTC by using agents that increase RAI uptake. Notably, TKIs may be particularly effective in triggering this enhancement. This therapy holds promise in addressing some of the challenges faced by patients with DTC. This review provides an overview of the therapies aimed at enhancing RAI incorporation, including those currently in clinical trials.

## Difference between ‘enhancing RAI incorporation’ and ‘redifferentiation’

The phrases ‘enhancing RAI incorporation’ and ‘resensitization to RAI’ are more specific than the term ‘redifferentiation’. Enhancing RAI incorporation or resensitization to RAI refers to therapies that promote RAI uptake in DTC cells by re-expressing functional sodium-iodide symporter (NIS) on cell membranes. In various references, ‘redifferentiation therapy’ in DTC conveys a similar concept. However, ‘differentiation’ is a broader term that encompasses the functional expression of the original cell, tumor growth potential and the degree of nuclear atypia. Often, the term differentiation is used to refer to only one aspect of this process. For example, in regenerative medicine, redifferentiation refers to the reacquisition of the functions of the original cell (21). In oncology, retinoic acid (RA) therapy for patients with neuroblastoma may be used to reduce tumor growth potential. In the thyroid gland, differentiation may refer to hormone-producing capacity or, in thyroid tumors, to tumor proliferative capacity. To avoid confusion, we have used the terms ‘enhanced RAI incorporation’ or ‘resensitization to RAI’ whenever possible in this study. However, the re-expression of NIS is also noted to be one of the outcomes of redifferentiation, and these processes are often interconnected and not easily distinguished.

## Molecular mechanism of RAI resistance

**Figure 1:**
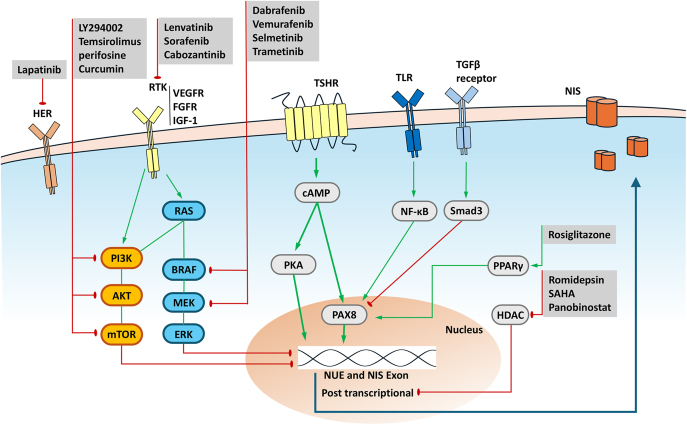
Overview of the regulation of NIS expression in DTC. cAMP, cyclic adenosine monophosphate; DTC, differentiated thyroid cancer; ERK, extracellular signal-regulated kinase; FGFR, fibroblast growth factor receptor; HDAC, histone deacetylase; IGF-1, insulin-like growth factor-1; HER, human epidermal growth factor receptor; mTOR, mechanistic target of rapamycin; NF-κB, nuclear factor kappa-light chain-enhancer of B cells; NIS, sodium-iodide symporter; NUE, NIS upstream enhancer; PAX8, paired box gene-8; PI3K, phosphatidylinositide-3-OH kinase; PKA, protein kinase A; PPARγ, peroxisome proliferator-activated receptor gamma; RTK, receptor tyrosine kinase; Smad3, Sma- and Mad-related protein 3; TGFβ, transforming growth factor β; TLR, toll-like receptor; TSHR, thyroid-stimulating hormone receptor; VEGFR, vascular endothelial growth factor receptor.

Iodide, a key component of thyroid hormones, is actively transported into thyroid follicular cells by NIS, a protein located on the basement membrane. The functional expression of NIS allows RAI to be selectively absorbed by the tumor, where it exerts its cytotoxic effect through intensive irradiation. While DTCs typically express NIS at the plasma membrane, progressive dedifferentiation results in reduced or dysfunctional NIS expression, which is a major cause of resistance to RAI therapy. In histopathology, RAI accumulation is prominent in well-differentiated FTC and the follicular variant of PTC. However, RAI accumulation tends to decrease as dedifferentiation progresses, with PDTC and anaplastic thyroid cancer (ATC) following PTC. This section outlines the mechanisms regulating NIS expression and the key signaling pathways involved (Fig. 1).

### Gene mutations and biological processes affecting DTC development and dedifferentiation

The Cancer Genome Atlas (TCGA) study reported that most PTCs harbor mutually exclusive driver gene mutations, primarily *BRAF^V600E^* and *RAS* gene mutations (described later), with some PTCs exhibiting *RET* fusions (22). A small subset of PTCs also contains *NTRK1*, *NTRK3* and *ALK* gene fusions, which are RTKs distinct from *RET*. In a review article, Landa *et al.* noted that FTC commonly presents with *RAS* mutations (25–30%), followed by *PAX8-PPARγ* fusions (approximately 25%) and mutations in *TSHR, PTEN* and *EIF1AX* (each occurring in 5–10% of cases) (23).

Several late-event mutations and biological processes are frequently identified in advanced thyroid cancers, such as ATC, PDTC and aggressively behaving DTCs (23). Abnormal activity of the phosphatidylinositide-3-OH kinase (PI3K)/AKT/mechanistic target of rapamycin (mTOR) pathway is driven by a high prevalence of mutations in *PIK3CA*, *AKT1* and *PTEN*. Moreover, the loss of p53 function, caused by *TP53* mutations, has been linked to the loss of PAX8 expression and impaired DNA repair mechanisms. Moreover, mutations in *RET*, *BRAF^V600E^*, *HRAS^G12V^*, *KRAS^G12D^*, *PTEN* and *STRN-ALK* have been reported to contribute to p53 dysfunction. *TERT* promoter mutations have been identified as genomic markers associated with thyroid cancer progression. Disruption of the SWI/SNF complex, playing a crucial role in chromatin remodeling, is also frequently observed in undifferentiated and poorly DTCs. Various other gene mutations contribute to the progression of thyroid cancer. For more detailed information, please refer to the review by Landa *et al.* (23).

### Mechanism of TSH receptor-associated NIS expression

Thyroid-stimulating hormone (TSH)-dependent and multiple TSH-independent pathways regulate functional NIS expression. TSH primarily acts at the post-translational level, upregulating NIS expression and promoting its trafficking to the plasma membrane. When TSH binds to its receptors on the plasma membrane, it increases cyclic adenosine monophosphate (cAMP) levels. cAMP activates the NIS upstream enhancer (NUE) through both protein kinase A (PKA)-independent (paired box gene-8, PAX8-mediated) and PKA-dependent (cAMP-response element-binding site-mediated) pathways, both critical for NUE’s integrative activity (24). PAX8 is regulated by three pathways, as outlined below. The pituitary tumor-transforming gene-1 (PTTG1) complex with its binding factor can disrupt PAX8 binding to NUE, suppressing NIS expression (25, 26). Transforming growth factor β (TGFβ) inhibits PAX8 via Sma- and Mad-related protein 3 (Smad3), significantly reducing *NIS* mRNA levels in thyroid cells (27, 28). Toll-like receptor (TLR) activates nuclear factor kappa-light chain-enhancer (NF-κB), which strengthens PAX8 signaling and stimulates NIS transcription through NUE (29, 30).

### Main signaling pathways and mutations affecting NIS expression

Several key signaling pathways influencing NIS expression have been elucidated. Mutations in genetic and epigenetic factors within the mitogen-activated protein kinase (MAPK) pathway, involving RAS/BRAF/MEK/ERK and PI3K signaling pathway are closely linked to dedifferentiation and suppression of NIS expression in DTC. These mutations can result from acquired point mutations, chromosomal rearrangements or aberrant gene methylation (31). The activation of these pathways also begins with the binding of growth factors to receptor tyrosine kinases (RTKs) such as the RET proto-oncogene, vascular endothelial growth factor receptor (VEGFR) and fibroblast growth factor receptor (FGFR) (32). Another RTK, the human epidermal growth factor receptor (HER) 2/3, can also reactivate these pathways (33). RTKs trigger MAPK and PI3K signaling through RAS (34), and some RTKs can directly activate the PI3K pathway as well.

*BRAF^V600E^* mutation is the most common genetic abnormality in thyroid cancer, present in nearly 50% of DTCs (35) and around 60% of PTCs. This point mutation aberrantly activates the MAPK pathways, correlating with reduced NIS expression and increased dedifferentiation, relapse and metastasis (27). BRAF activation represses NIS expression through two major mechanisms. First, BRAF activates TGFβ and Smad3 signaling, leading to a dose-dependent reduction in PAX8 DNA-binding activity, along with decreased NIS mRNA and protein levels (27, 28). Second, the *BRAF^V600E^* mutation promotes histone deacetylation of the NIS promoter, impairing NIS expression (36). MEK, downstream of BRAF, also directly affects NIS expression (37). Combined targeting of BRAF and MEK has demonstrated enhanced MAPK inhibition in preclinical models, resulting in more pronounced NIS expression (38).

The differences between *BRAF^V600E^* and *RAS* mutations can be attributed to the distinct signaling patterns observed in *BRAF^V600E^*- and *RAS*-like PTCs in TCGA (22). The MAPK and PI3K signaling pathways are differentially activated in these tumor types. In *BRAF^V600E^* mutation cases, MAPK signaling is strongly activated, resulting in an increased output of the ERK transcriptional program because of the resistivity of *BRAF^V600E^* mutation to ERK inhibitory feedback. By contrast, the MAPK and PI3K/AKT/mTOR pathways are activated in *RAS*-like tumors, in which PI3K signaling primarily occurs through CRAF, which remains susceptible to ERK feedback inhibition. These signaling differences suggest that thyroid differentiation and NIS expression are better preserved in *RAS*-mutant tumors than in *BRAF^V600E^*-mutant tumors.

Several reports indicate that activation of the PI3K/AKT/mTOR signaling pathway inhibits NIS transcription (39, 40). Various growth factors, including insulin/insulin-like growth factor-1 (IGF-1) and epidermal growth factor, can activate the PI3K pathway (39). PI3K is directly involved in regulating NIS expression. The AKT inhibitor curcumin has been shown to increase NIS glycosylation and membrane trafficking (41). Downstream, mTOR regulates cell metabolism and proliferation, and both *in vitro* and *in vivo* studies have demonstrated that mTOR inhibition not only controls cell survival but also enhances RAI uptake (40). In addition, mTOR has been implicated in NIS expression via thyroid transcription factor-1 (TTF1) *in vitro* (42).

## Definition of RAIR-DTC

The variation in therapeutic efficacy, AEs and national regulatory policies regarding RAI therapy for advanced DTC, combined with its slow clinical progression and the emergence of next-line treatment options, often complicates determining the optimal timing to shift from RAI treatment to subsequent therapies. Before the advent of MKIs, determining RAI refractoriness was less accepted due to the lack of effective post-RAI therapies. However, the implementation of phase III trials assessing the efficacy of lenvatinib and sorafenib in advanced DTC led to the broader recognition of RAI refractoriness (14, 15). With the increasing availability of drugs such as lenvatinib and sorafenib for post-RAI treatment, it is expected that the growing range of options will further refine the definition of RAI refractoriness. Commonly accepted criteria for RAIR tumors now include i) no RAI uptake in local recurrence or distant metastases on a diagnostic ^131^I scan; ii) no or gradual loss of RAI uptake on a post-treatment scan; iii) RAI uptake in some tumor foci but not others; iv) structural tumor progression 12–16 months after RAI treatment despite RAI uptake; v) tumors in patients who have received >600 mCi (22.2 GBq) cumulative RAI with no signs of remission; and vi) significant uptake on ^18^F-fluorodeoxyglucose (^18^F-FDG) positron emission tomography/computed tomography (PET/CT). A joint statement from the American Thyroid Association, the European Association of Nuclear Medicine, the Society of Nuclear Medicine and Molecular Imaging and the European Thyroid Association emphasizes that none of the criteria for determining RAI refractoriness should be viewed in isolation as definitive. Instead, these criteria should be used to stratify patients based on the likelihood that their tumor will respond to RAI therapy. Risk stratification may also account for histologically aggressive or poorly differentiated tumors, and those with aggressive genetic profiles, such as *BRAF* or *TERT* promoter mutations (43, 44). Van Nostrand (45) suggests that the criteria for determining RAI-refractory disease will continue to evolve as physicians better understand the limitations and confounding factors of current classifications and as new agents that increase or restore RAI uptake become available. The criteria for RAI resistance may also be further refined if enhancing RAI incorporation becomes a standard approach for advanced DTC. For instance, in cases where enhancing RAI incorporation is planned, the 600 mCi (22.2 GBq) threshold in criterion (v) might be adjusted downward, taking into account the cumulative dose. Establishing appropriate thresholds for when to introduce novel therapies remains a significant clinical challenge, necessitating further discussion to optimize treatment strategies.

## Enhancing RAI incorporation in RAIR-DTC

Enhancing RAI incorporation aims to restore the effectiveness of RAI therapy for patients with RAIR-DTC by using agents that can potentially re-express NIS on the plasma membrane and increase tumor RAI uptake. Previous studies have demonstrated that RAI-positive DTC is associated with a better prognosis than RAI-negative disease. Moreover, as outlined below, many of the drugs that may improve RAI uptake are also antitumor agents. Beta rays, known for their efficient cytotoxic effects on small tumors, may enhance the therapeutic impact of RAI therapy when combined with tumor shrinkage induced by these agents. This section will review past research on enhancing RAI incorporation and discuss ongoing clinical trials. Supplementary Table 1 (see section on Supplementary materials given at the end of the article) summarizes the results of previously reported prospective trials, while Supplementary Table 2 summarizes the ongoing trials exploring strategies to enhance RAI incorporation.

### RA

RA was initially explored for resensitizing DTC to RAI, but several prospective studies have shown that RA monotherapy does not significantly enhance RAI uptake in DTC. RA, a derivative of vitamin A, influences mRNA transcription factors and exhibits strong anti-proliferative and differentiation-inducing properties, rendering it a candidate for redifferentiation therapy and cancer chemoprevention. Although basic research has demonstrated potential benefits (46), retrospective cohort studies have reported mixed results regarding ability of RA to improve tumor RAI uptake (47, 48, 49). Some studies reported no clinically meaningful improvement in RAI uptake (50), and the findings have been inconsistent. A randomized phase II trial by Short *et al.* found no significant resensitization to RAI in most cases (51). Similarly, two other prospective studies reported that only two of eleven cases of RAIR-DTC showed slight improvement in RAI uptake (52, 53). Based on these prospective studies, RA alone appears unlikely to enhance RAI uptake in patients with metastatic DTC. However, Groener *et al.* (54) reported that RA improved RAI uptake in a small cohort of patients with RAIR-DTC harboring *BRAF^V600E^* mutations, suggesting that redifferentiation potential of RA may be effective in specific clinical contexts.

### PPARγ

Peroxisome proliferator-activated receptors (PPARs) are ligand-activated transcription factors, and several preclinical studies suggest that PPARγ agonists may aid in RAI uptake in DTC. Fröhlich *et al.* (55) demonstrated that the PPARγ agonist troglitazone increased ^125^I uptake and NIS expression in normal porcine thyroid cells and follicular cancer cell lines when combined with RA. In a study involving six human thyroid cancer cell lines, Park *et al.* (56) found that troglitazone downregulated the surface expression of CD97, a novel dedifferentiation marker, in FTC-133 cells and upregulated NIS mRNA in TPC-1 and FTC-133 cells. Moreover, Chen *et al.* (57) reported that rosiglitazone inhibited cell proliferation and increased NIS protein expression in human thyroid cancer cells.

To date, only one clinical trial is reported evaluating the resensitizing effect of PPARγ agonists. In an open-label phase II study, Kebebew *et al.* (58) assessed the rate of RAI resensitization and the therapeutic effect of rosiglitazone in 20 patients with DTC that was either refractory to RAI uptake or unresectable. Following rosiglitazone treatment, 25% (five out of 20) of the patients had a positive RAI scan. However, none of the patients achieved a CR or partial response (PR) based on RECIST criteria at the 3-month follow-up, indicating that rosiglitazone alone had limited therapeutic efficacy. Currently, no clinical trials investigating this approach are ongoing.

### HDAC inhibitors

Histone acetylation plays a crucial role in the epigenetic regulation of gene expression and is often hyperactive in advanced thyroid cancers, including various carcinomas. Histone deacetylase inhibitors (HDACIs) exhibit potent anticancer properties, considerably affecting cell viability and differentiation. In several imaging experiments and *in vivo* studies (59, 60, 61), thyroid tumor cells treated with HDACIs demonstrated increased radioiodine uptake and specific accumulation of RAI.

Unfortunately, none of the prospective clinical trials have shown a clear improvement in iodine uptake with HDACI treatment. Sherman *et al.* (62) evaluated the response rate of romidepsin, an HDACI, and changes in RAI avidity in a Simon two-stage phase II clinical study involving patients with RAIR-DTC. The study found that only two of the 20 patients exhibited RAI reuptake in their tumors. Similarly, in a phase I study of romidepsin involving patients with thyroid cancer, Amiri-Kordestani *et al.* (63) reported that follow-up RAI scans in patients with RAIR-DTC showed no meaningful increases in uptake (zero of the six patients). Moreover, in an open-label phase II trial of valproic acid, none of the ten patients who completed 10 weeks of treatment demonstrated increased tumor RAI uptake (64). These findings suggest that HDACI monotherapy is unlikely to sufficiently resensitize RAI uptake for effective RAI retreatment.

### MAPK inhibitors

Inhibiting the MAPK signaling pathway with drugs such as BRAF and/or MEK inhibitors is a central focus of preclinical and clinical research on enhancing RAI incorporation. Several clinical trials have shown promising RAI resensitization rates for clinical use. Ongoing trials, as discussed below, also emphasize MAPK inhibitors. This section summarizes the relevant clinical trials.

Several preclinical studies have demonstrated that targeting MAPK signaling pathways may enhance the expression of thyroid iodide-metabolizing genes and increase RAI uptake in tumor cells. Liu *et al.* (37) found that the MEK inhibitor U0126, or cessation of BRAF^V600E^ expression in PCCL3 cells, restored the expression of thyroid genes previously silenced by *BRAF^V600E^*. U0126 also restored these gene expressions in *BRAF^V600E^*-harboring, PTC-derived NPA cells. Hou *et al.* (65) reported that the MEK inhibitor RDEA119, mTOR inhibitor temsirolimus, AKT inhibitor perifosine and histone deacetylase inhibitor SAHA each restored the expression of iodide-handling genes such as NIS, TSH receptor and thyroperoxidase in thyroid cancer cell lines. A study using the MEK inhibitor selumetinib in thyroid cells and *BRAF^V600E^*-induced thyroid cancer in mice (38) showed that MEK inhibitors suppress ERK signaling. Even modest ERK inhibition increased the expression of thyroid differentiation genes and iodide accumulation in cancer cells, improving their response to RAI therapy. In addition, the study demonstrated that combining the RAF inhibitor PLX4032 with U0126 led to even stronger NIS expression in PCCL3 cells.

Ho *et al.* (66) reported on the resensitization of tumors to RAI in patients with metastatic thyroid cancer using selumetinib (AZD6244, ARRY-142886) (ClinicalTrials.gov identifier NCT00970359). They assessed resensitization by performing ^124^I PET dosimetry before and after administering selumetinib (75 mg twice daily for 4 weeks). Selumetinib increased ^124^I uptake in 12 of the 20 patients (four of nine with *BRAF* mutations and all five with *NRAS* mutations). Of these 12 patients, eight reached the dose threshold for RAI therapy and received it. Among these eight, five showed a confirmed PR and three had stable disease (SD), with serum thyroglobulin levels decreasing in all patients (mean reduction of 89%). No grade 3 or higher toxicities were attributed to selumetinib, although one patient developed acute leukemia more than 51 weeks after RAI treatment.

In a prospective clinical trial (NCT01534897), Rothenberg *et al.* (67) assessed the efficacy of resensitization using the BRAF inhibitor dabrafenib in ten patients with iodine-resistant PTC harboring the *BRAF^V600E^* mutation. Patients received dabrafenib (150 mg twice daily) for 25 days. Patients exhibited new radioiodine uptake on scans continued dabrafenib for an additional 17 days and then underwent RAI therapy with 150 mCi (5.6 GBq). In total, six out of ten patients (60%) showed new radioiodine uptake on whole-body scans after dabrafenib treatment. Of these, two had a PR and four had SD at 3 months post-RAI therapy. Tg levels decreased in four of the six patients. One patient developed squamous cell carcinoma of the skin, but no other serious dabrafenib-related AEs were observed. In another prospective trial by Dunn *et al.* (68), four of ten evaluable patients responded to the BRAF inhibitor vemurafenib with ^124^I uptake and subsequent RAI therapy led to tumor regression after 6 months.

In a prospective, single-center, two-arm phase II trial (ERRITI trial, ClinicalTrials.gov ID NCT04619316) (69), with the primary endpoint of RAI resensitization rate, patients with RAIR-DTC harboring either a *BRAF* mutation or *BRAF*-WT were treated with trametinib (*BRAF*-WT) or trametinib plus dabrafenib (*BRAF* mutation) for 21 ± 3 days. RAI uptake increased in seven of 20 patients (35%), with two of six (33%) in the *BRAF* mutation group and five of 14 (36%) in the *BRAF*-WT group. Seven patients received subsequent RAI therapy (mean activity 300 mCi (11.1 GBq)). Thyroglobulin levels decreased in 57% (4/7) of patients, and RECIST 1.1 assessments showed SD/PR/PD rates of 71% (5/7), 14% (1/7) and 14% (1/7), respectively. One patient experienced transient grade 3 fever, and another had a grade 4 rash. These and other pilot and prospective trials suggest the efficacy and safety of targeting the MAPK signaling pathway for RAI resensitization using BRAF and MEK inhibitors. A key advantage of these agents is the ability to obtain RAI uptake with short-term treatment, making AEs that would be unacceptable with long-term use more tolerable. Further studies are anticipated to optimize protocols, evaluate long-term efficacy and better understand side effects and clinical outcomes.

Interestingly, previous clinical studies have suggested that inhibiting this pathway in patients with *RAS* mutation-positive tumors may result in an exceptionally high rate of tumor resensitization to RAI. As mentioned earlier, in a prospective trial of selumetinib (AZD6244, ARRY-142886) by Ho *et al.* (66), all five patients with *RAS* mutations regained RAI uptake using selumetinib. In addition, a recent retrospective cohort study showed that all eleven patients with *RAS* mutation-positive RAIR-DTC treated with MEK, RET, or ALK inhibitors, either alone or with a BRAF-MEK combination, achieved RAI avidity (70). Future studies on *RAS* gene mutations in thyroid cancer are warranted (71).

### HER2/3 inhibitors with MAPK inhibitors

HER2/3, members of the RTK family, may contribute to radioresistance in DTC. BRAF/MEK inhibitors for RAIR-DTC often lead to a rebound in MAPK signaling. Preclinical studies have shown that this ERK rebound in thyroid cells is accompanied by increased HER3 signaling, suggesting that HER2/HER3 activation plays a role (33). Garcia-Rendueles *et al.* (72) reported that resistance to vemurafenib in *BRAF*-mutant thyroid cells was driven by HER2 and HER3 activation across all tested isogenic human and mouse thyroid cell lines, and this resistance was mitigated by pan-HER kinase inhibitors. In another *in vitro* experiment, combining the HER inhibitor lapatinib with BRAF/MEK inhibitors dabrafenib/selumetinib significantly enhanced redifferentiation in *BRAF^V600E^*-mutant papillary thyroid cancer cells compared to BRAF/MEK inhibition alone (73). Supportive results were also observed in a pilot study. Tchekmedyian *et al.* (74) validated RAI resensitization using vemurafenib combined with CDX-3379, a HER3 inhibitor. In this study, RAI uptake increased in five of six patients with *BRAF^V600E^*-mutant thyroid cancer, leading to RAI treatment in four. There were no grade 3 or 4 toxicities linked to CDX-3379. At 6 months, two patients achieved PR, while two had PD (ClinicalTrials.gov: NCT02456701). These results suggest that further evaluation of HER3 inhibitors for enhancing RAI incorporation in larger trials is warranted.

### MKI

Preclinical studies suggest that MKIs have the potential to resensitize tumors to RAI, although this efficacy has not been confirmed in clinical trials. A key advantage of MKIs is their applicability across RAIR-DTC cases, regardless of specific genetic mutations. MKIs target multiple points in the MAPK and PI3K pathways, including VEGFR 1–3, PDGFR, RET, KIT proto-oncogene and BRAF (14, 15). Theoretically, MKIs should contribute to RAI resensitization in RAIR-DTC. A preclinical study using mice with ATC xenografts demonstrated that the TKI K905-0266 increased endogenous NIS expression and enhanced the therapeutic effect of RAI (75). Sorafenib and lenvatinib, two FDA-approved drugs for RAIR-DTC, are central to ongoing clinical research. In a prospective phase II trial involving 31 patients with RAIR-DTC, sorafenib was tested for its ability to resensitize tumors to RAI (76). However, none of the patients showed increased RAI uptake at metastatic sites on scintigrams after 26 weeks of sorafenib (400 mg twice daily), suggesting sorafenib’s ineffectiveness in improving RAI uptake. A RESET trial evaluating another MKI, lenvatinib, is currently underway (ClinicalTrials.gov number, NTC04858867) (77).

### PI3K inhibitors

The PI3K/AKT/mTOR signaling pathway has been primarily studied preclinically using inhibitors targeting each part of the pathway. The PI3K inhibitor LY294002 increased radioiodine uptake, mainly by enhancing NIS expression *in vitro* (78, 79). mTOR inhibitors had a milder effect on RAI uptake compared to PI3K inhibition (40, 42), but did increase thyroid iodide uptake in rats *in vivo* (42). The AKT inhibitor curcumin improved NIS glycosylation and membrane transport, significantly enhancing RAI uptake in an *in vitro* study (41). These preclinical studies suggest that various points in this pathway influence RAI uptake in thyroid cancer cells. Further investigation will be necessary to assess the clinical benefits of PI3K/AKT/mTOR inhibitors for enhancing RAI uptake in patients.

### Combinations: RA, PPARγ and HDACI

As discussed, the use of RA, PPARγ agonists and HDACIs as single agents has not shown sufficient success in clinical trials to achieve significant RAI uptake. However, recent preclinical studies suggest that combining these agents with other sensitizers may yield a more substantial increase in RAI uptake. Chen *et al.* (57) reported that rosiglitazone reduced cell growth and increased NIS protein expression in human thyroid cancer cells, with the retinoid X receptor agonist bexarotene enhancing these effects. Hou *et al.* (65) validated the impact of various inhibitors – RDEA119 (MAPK inhibitor), temsirolimus (mTOR inhibitor), perifosine (AKT inhibitor) and SAHA (HDACI) – on iodine-handling gene expression and RAI uptake in thyroid cancer cell lines. Their study demonstrated that these inhibitors, particularly when combined with SAHA, robustly and synergistically restored the expression of iodine-handling genes such as NIS, TSH receptor and thyroperoxidase. This suggests that HDACIs could enhance the effects of therapies targeting the MAPK and PI3K pathways. In another study using *BRAF^V600E^*-mutant (BCPAP and K1) and *BRAF* wild-type (BHP 2–7) cells, combination therapy with panobinostat and MAPK inhibitors (dabrafenib or selumetinib) produced a stronger *BRAF^V600E^*-dependent repopulation effect (80). Future clinical trials are needed to confirm these drug combinations’ efficacies in enhancing RAI uptake and to evaluate their toxicity profiles.

### Ongoing clinical trial of enhancing RAI incorporation

As of June 2024, eleven clinical trials registered on ClinicalTrials.gov are ongoing to evaluate the efficacy of enhancing RAI incorporation (Supplementary Table 2). These include two pilot studies, seven phase II trials, one phase I trial and one observational study involving ^124^I PET/CT. Results from the primary trials NCT02145143 and NCT04619316 have been published (68, 69). These trials are ongoing, and expansion studies are anticipated.

These trials may address some uncertainties surrounding the enhancement of RAI incorporation. One key issue is optimizing therapeutic dosing, which remains controversial. Van Nostrand *et al.* suggested in a review that dosimetry is crucial for enhancing RAI incorporation (81). NCT06443866 is using ^124^I PET/CT to evaluate changes in RAI uptake before and after therapeutic intervention in recurrent/metastatic DTC. Past and ongoing clinical trials can be divided into two categories based on therapeutic dose: those with a fixed dose of 150 mCi (5.6 GBq) and those where the tumor dose is set above a certain threshold. These trials may provide a clearer basis for determining the optimal dose in the future. Another important question is when to introduce RAI incorporation enhancement. The NCT06440850 trial includes patients with RAI-naive and high-risk thyroid cancer, meaning enhancement is introduced before confirming RAIR-DTC status. Previous studies have shown that the cost and side effects of MAPK inhibitors are limited due to their short duration, typically around 1 month. If RAI incorporation enhancement proves highly effective, early intervention may be justified, even before RAIR-DTC determination. In addition, the double-blind, randomized-controlled design of the NCT02393690 trial may provide data on OS. Along with therapy response and AEs, determining the optimal threshold and timing for enhancing RAI incorporation is crucial for developing the best treatment strategy for DTC.

## Predictors of enhancing RAI incorporation

Targeted therapies often lead to RAI resensitization in DTC, even during standard use, without specific protocols for enhancing RAI incorporation. Therefore, understanding the factors that predispose patients to resensitization and identifying changes in clinical findings when RAI incorporation is enhanced is essential. The ERRITI study (69) identified predictors of successful RAI resensitization, finding that a peak standardized uptake value (SUVpeak) <10 on FDG-PET was associated with successful resensitization (*P* = 0.01). A pilot study by Dunn *et al.* reported that the mean pretreatment serum Tg was higher in the response group compared to the nonresponse group (30.6 vs 1.0 ng/mL; *P* = 0.0048) (68). Montes de Jesus *et al.* also demonstrated that divergence between increased Tg and structural response could be a reliable biomarker for RAI resensitization (82). In such cases, a diagnostic scan with ^123^I or ^124^I could be considered for additional RAI therapy. In a retrospective study, Jaber *et al.* (83) described 13 patients with RAIR-DTC who received TKIs and subsequently showed RAI uptake, enabling RAI therapy. The patients had received BRAF or MEK inhibitors for a median of 14.3 months before resensitization. Nine (69%) patients underwent RAI therapy (median dose, 204.4 mCi (7.6 GBq)), after which they discontinued BRAF or MEK inhibitors. All nine achieved durable disease control (3/9 PR, 6/9 SD). AEs included pneumonitis and sialadenitis.

Based on the results of previous clinical trials (66, 70), we speculate that enhancing RAI incorporation for RAIR-DTC with *RAS* mutations offers substantial benefits because of the relatively preserved thyroid differentiation and NIS expression characteristic of the original *RAS*-like tumors. Even in cases involving the *BRAF^V600E^* mutation, the combination of BRAF and MEK inhibitors can provide some benefits. However, the effect of RAI resensitization may be considerably reduced in the presence of multiple genetic mutations. Saqcena *et al.* (84) proposed that the loss of the SWI/SNF complex leads to RAI refractoriness and resistance to resensitization based on MAPK inhibitors in *BRAF^V600E^*-mutant PTC in mice. In addition, Syed *et al.* (85) reported that concomitant *TERT* promoter mutations in the *NTRK* fusion gene may hinder resensitization to RAI, as demonstrated through the clinical course of two model patients. Several studies have shown that the combination of *TERT* promoter and *BRAF^V600E^* mutations is strongly linked to the loss of RAI avidity in PTC (86, 87). We speculate that the combination of these genetic variants is likely to be a poor predictor of enhancing RAI incorporation. Identifying factors that resist improving RAI uptake remains a critical clinical challenge.

## Limitations

Despite the promising potential of enhancing RAI incorporation, several limitations in current studies must be acknowledged. First, the sample size and the number of clinical trials conducted are small, limiting the generalizability of findings. Second, significant challenges related to RAI scans are observed. Only a limited number of facilities can utilize isotopes such as ^123^I, ^124^I and ^131^I. In particular, very few centers are equipped to perform ^124^I-PET scans, which allow for precise quantification. Moreover, quantifying single-photon scans using ^123^I and ^131^I remains difficult. Further complicating this approach are issues related to clinical protocols and treatment courses. The criteria for defining RAIR-DTC, the optimal timing for RAI resensitization, and the most effective definition of improved RAI uptake have yet to be established. Moreover, clear differentiation between the effects of antitumor drugs used for RAI resensitization and the direct effects of RAI therapy is challenging. For this strategy to be deemed effective, it must result in either an anatomical or biochemical response. In addition, currently, no evidence exists to guide whether antitumor drugs should be continued when RAI resensitization. No country has approved the use of recombinant human thyrotropin in combination with RAI treatment for distant metastatic DTC. This regulatory hurdle must be addressed before enhancing RAI incorporation can move from experimental trials into standard clinical practice. In this way, we must recognize that many problems remain unresolved.

## Conclusion

NIS expression on tumor cell membranes is why RAI remains a promising therapy for advanced DTC. However, various genetic mutations in DTC can reduce tumor NIS expression and lead to RAI resistance, primarily through activation of the MAPK signaling pathway. TKIs targeting this pathway have shown promising efficacy in clinical trials for enhancing RAI incorporation. In the treatment of RAIR-DTC, enhancing RAI incorporation could address the challenges and limitations of current therapies. At the same time, we also need to recognize that many challenges remain with this new treatment approach. We look forward to further mechanistic studies and the outcomes of ongoing clinical trials in these patient populations.

## Supplementary materials



## Declaration of interest

The authors declare that there is no conflict of interest that could be perceived as prejudicing the impartiality of the work reported.

## Funding

The research did not receive any specific grant from any funding agency in the public, commercial or not-for-profit sector.
