# Supplementary material for: Enhancing radioactive iodine (RAI) incorporation in RAI-refractory differentiated thyroid cancer: current insights
Source: Eur Thyroid J. 2025 Mar 24;14(2):e240319. doi: 10.1530/ETJ-24-0319 (PMC11964482; doi:10.1530/ETJ-24-0319)
Supplement: Supplementary file 1 [file supplementary_materials.pdf]

Supplementary Table 1. Summary of clinical trials evaluating the enhancement of radioactive iodine (RAI) incorporation in patients with differentiated thyroid cancer (DTC).

NCT number	Year	Author	Title	Design	Inclusion	Evaluated	Histopathology	Mutation	Intervention	Success rate	Response to enhancing RAI incorporation
NCT00887107	2009	Hoftijzer, H.	Beneficial Effects of Sorafenib on Tumor Progression, but Not on Radioiodine Uptake, in Patients with Differentiated Thyroid Carcinoma-Arm Study	Single-center Single-arm Phase II	31	31	DTC	None	Sorafenib	0/31	No additional RAI treatment
NCT00048334	2013	Amiri-Kordestani, L.	Phase I Trial of a New Romidepsin in Patients with Advanced Cancers	Phase I	11	6	3 PTC<br>3 FTC	None	Romidepsin	0/6	No additional RAI treatment
NCT00970359	2013	Ho, A. L.	Selumetinib Enhances Radioiodine Uptake in Advanced Thyroid Cancer	Single-center Single-arm Pilot study	24	20	None	9 *BRAF*<br>5 *NRAS*<br>3 *RET*/*PTC*<br>3 wild type	Selumetinib	12/20<br>4/9 *BRAF*<br>5/5 *NRAS*<br>2/3 *RET*/*PTC*<br>1/3 wild type	No additional RAI treatment
NCT01534897	2015	Rothenberg, S. M.	Redifferentiation in Iodine-Refractory BRAF V600E-Mutant Metastatic Papillary Thyroid Cancer	Single-center Single-arm	10	10	PTC	$BRAF^{V600E}$	Dabrafenib	6/10	RECIST: 2/6 PR, 4/6 SD<br>Tg decrease: 4/6
NCT02145143	2019	Dunn, L. A.	Vemurafenib Redifferentiation of BRAF Mutant, RAI-Refractory Thyroid Cancers	Single-center Single-arm Pilot study	12	10	9 PTC<br>3 PDTC	$BRAF^{V600E}$	Vemurafenib	4/12	2/4 PR, 2/4 SD

NCT03244956	2021	Leboulleux, S	MERAIODE: A Redifferentiation Phase II Trial with Trametinib and Dabrafenib Followed by Radioactive Iodine Administration for Metastatic Radioactive Iodine Refractory Differentiated Thyroid Cancer Patients with a BRAFV600E Mutation	Multi-center Non-randomized Phase II	24	21	21 PTC	*BRAF*^{V600E}	Dabrafenib and trametinib	11/17	8/21 responder
NCT02456701	2022	Tchekmedyian, V.	Enhancing Radioiodine Incorporation in BRAF-Mutant, Radioiodine-Refractory Thyroid Cancers with Vemurafenib and the Anti-ErbB3 Monoclonal Antibody CDX-3379: Results of a Pilot Clinical Trial	Single-center Single-arm Phase I	7	6	DTC, except for PDTC	*BRAF*^{V600E}	Vemurafenib + CDX-3379	5/6	2/4 PR, 2/4 PD
NCT04619316	2022	Weber, M.	Enhancing Radioiodine Incorporation into Radioiodine-Refractory Thyroid Cancer with MAPK Inhibition (ERRITI): A Single-Center Prospective Two-Arm Study	Single-center Non-randomized Phase II	20	20	10 PTC<br>7 FTC<br>3 PDTC	6 *BRAF*^{V600E}<br>14 *BRAF*-WT	Trametinib (*BRAF*-WT) trametinib + dabrafenib (*BRAF*^{V600E})	2/6 *BRAF*^{V600E}<br>5/14 *BRAF*-WT	1/7 PR, 5/7 SD, 1/7 PD<br>4/7 Tg decline

Supplementary Table 2**.** Ongoing clinical trials registered on ClinicalTrials.gov evaluating RAI incorporation enhancement.

NCT number	Title	Study design	Enrollment (Estimated)	Main assessment	Patients	Mutation	Intervention agents
NCT04858867	Reinducing Radioiodine-Sensitivity in Radioiodine-refractory DTC Using Lenvatinib (RESET)	Single-center Open label Non-randomized Phase II	12	Redifferentiation effect	RAIR-DTC	None	Lenvatinib
NCT05182931	A Prospective, Multi-Centre Trial of TKI Redifferentiation Therapy in Patients with RAIR Thyroid Cancer (I-FIRST Study)	Multi-center Open label Non-randomized Phase II	80	PFS, OS, AE, ORR, and quantification of radioiodine uptake in metastatic lesions before and after TKI treatment	RAIR-DTC	*NRAS* or $BRAF^{V600E}$	Trametinib (for *NRAS*) or combination of trametinib and dabrafenib (for $BRAF^{V600E}$)
NCT04619316	Enhancing Radioiodine Incorporation into Radio Iodine Refractory Thyroid Cancers with MAPK Inhibition (ERRITI)	Single-center Non-randomized Phase II	70	Proportion of patients who achieve increased iodine incorporation	RAIR-DTC	*BRAF*-WT and $BRAF^{V600E}$	Trametinib (for WT) or combination of dabrafenib and trametinib (for $BRAF^{V600E}$)
NCT02145143	Enhancing Radioiodine (RAI) Incorporation into BRAF Mutant, RAI-Refractory Thyroid Cancers with the BRAF Inhibitor Vemurafenib: A Pilot Study	Single-center Open label Pilot study	12	ORR, AE, and proportion of patients who achieve increased iodine incorporation	RAIR-DTC	$BRAF^{V600}$	Vemurafenib
NCT02152995	Trametinib in Increasing Tumoral Iodine Incorporation in Patients With Recurrent or	Single-center Open label Non-randomized	34	OS, ORR (6 m), and proportion of patients who achieve increased	RAIR-DTC	*RAS* or *RAS*/*RAF*-WT	Trametinib

	Metastatic Thyroid Cancer	Phase II		tumoral iodine incorporation			
NCT06440850	Vemurafenib and Cobimetinib for the Treatment of Patients with High Risk Differentiated Thyroid Carcinoma With BRAFV600E Mutation	Open label Phase II Pilot study	21	Proportion of patients who achieve increased iodine incorporation	High risk DTC (ATA risk category)	*BRAF*^{V600E}	Vemurafenib and Cobimetinib
NCT02393690	Iodine I-131 with or Without Selumetinib in Treating Patients with Recurrent or Metastatic Thyroid Cancer	Randomized Double-blind Phase II	60	ORR, PFS, OS, and AE	Recurrent or metastatic DTC, RAI-avid lesion on a radioiodine scan performed ≤24 months	None	Selumetinib
NCT04952493	Anlotinib or Penpulimab in Combination With RAI for DTC	Randomized Open-label Phase Il	42	ORR, BRR, PFS, and proportion of patients who achieve increased iodine incorporation, FDG uptake	Local advanced or metastatic DTC absence of good remission of RAI or may not get satisfactory remission from RAI treatment	None	Anlotinib hydrochloride and Penpulimab
NCT03469011	A Study to Try to Bring Back Radioiodine Sensitivity in Patients With Advanced Thyroid Cancer	Dose escalation Phase I	18	AE, restore iodine uptake, and Tg levels and anatomic imaging	RAIR-DTC	None	Imatinib
NCT05507775	Digoxin for the Reinduction of Radioiodine Uptake in Metastatic or Locally Advanced Non-medullary Thyroid Carcinoma (DIGUP-TC)	Single-center Open label Non-randomized Pilot study	10	Reinduction of radioiodine uptake, ORR (6 m), and AE	RAIR-DTC	None	Digoxin
NCT06443866	I-124 PET/CT Imaging and Dosimetry for RAI-Naïve or	Observational	50	Dosimetry	Post-total thyroidectomy patients with diagnosis of thyroid cancer, any	Any	Any

Refractory Thyroid Cancer	histology, subtype, ATA risk category, for evaluation for residual disease, cervical, or remote metastatic disease
